# Evaluation of a Tablet-Based Emotion Regulation Intervention for Surrogate Decision-Makers of Patients With Critical Illness: Pilot Nonrandomized Trial

**DOI:** 10.2196/73769

**Published:** 2026-01-19

**Authors:** Grant Pignatiello, Paul J Tuschman, Stephanie Alisha Griggs, Nicholas K Schiltz, Heath A Demaree, Alex Klinck, Alan Hoffer, Ronald L Hickman Jr

**Affiliations:** 1 Frances Payne Bolton School of Nursing Case Western Reserve University Cleveland United States; 2 Department of Psychological Sciences Case Western Reserve University Cleveland, OH United States; 3 Department of Neurological Surgery Harrington Heart and Vascular Institute University Hospitals of Cleveland Cleveland, OH United States; 4 School of Medicine Case Western Reserve University Cleveland, OH United States

**Keywords:** coping, critical care, emotion regulation, intensive care unit, shared decision-making

## Abstract

**Background:**

Psychological distress among surrogate decision-makers (surrogates) for patients with critical illness is well documented. Existing interventions for supporting surrogates in their role often target surrogates’ informational needs without directly addressing surrogates’ acute emotional burden. Therefore, we developed the Reappraisal-Enhanced Foundation for Regulating Affect and Managing Emotions (REFRAME) intervention, a tablet-based app that empowers surrogates to manage their psychological distress with cognitive reappraisal.

**Objective:**

We sought to (1) determine the feasibility, acceptability, and appropriateness of implementing REFRAME and (2) examine its preliminary effects on surrogates’ psychological distress.

**Methods:**

We conducted a pilot nonrandomized trial at a tertiary medical center in northeast Ohio. We recruited adult surrogates for incapacitated intensive care unit (ICU) patients (≥48 hours). The first 20 participants received usual care (UC); the next 28 received UC and REFRAME, consisting of 3 sequential 10- to 15-minute modules administered every 24 to 48 hours (T1-T3) post enrollment (T0). We evaluated implementation outcomes both quantitatively and qualitatively by describing enrollment and completion rates, surrogates’ scores on the Acceptability of Intervention Measure and the Intervention Appropriateness Measure, and thematically analyzing feedback from each interventional module. We measured psychological distress with the Patient-Reported Outcomes Measurement Information System Anxiety and Depression short forms at enrollment (T0) and approximately 1-week post enrollment (T3). We used linear mixed-effects models to assess changes in anxiety and depression severity between groups from T0 to T3, adjusting for the surrogate’s gender, patient relationship, prior decision-making experience, and perceived stress.

**Results:**

Our analytic sample included 48 surrogates (UC=20; REFRAME=28). Two-thirds (19/28, 67.9%) of those assigned to REFRAME completed all 3 modules, with over 70% finding it acceptable and appropriate. Qualitative feedback indicated that surrogates appreciated the intervention’s normalization of their emotions and provision of practical reappraisal strategies. Both groups showed reductions in psychological distress severity, with greater reductions in depressive symptoms reported by surrogates in the REFRAME group (*d*=0.68).

**Conclusions:**

REFRAME was feasible to implement, well-received by users, and considered relevant in the ICU setting. We observed preliminary improvement in depressive symptoms, though the effects on anxiety are less certain. Our findings indicate that incorporating brief cognitive reappraisal tools into routine ICU practice may support surrogates’ psychological well-being. Larger, more diverse trials with longer follow-up are necessary to confirm these initial findings and assess their impact on shared decision-making.

## Introduction

Psychological distress is a significant challenge for surrogate decision-makers (hereafter, surrogates) in the intensive care unit (ICU), with more than 50% reporting symptoms of anxiety and depression [1,2]. This distress is associated with impairments in sleep, cognition, and judgment that diminish their capacity to make value-concordant decisions for the patient [3-8]. Left unaddressed, psychological distress may contribute to nonbeneficial, value-discordant care and increase surrogates’ risk of long-term psychiatric morbidity [2,9-11].

Over the last 4 decades, many supportive interventions have been developed to address surrogates’ psychological needs. However, these interventions show limited and unreliable effectiveness in reducing both short- and long-term psychological distress [12-15]. Most prioritize surrogates’ informational needs by implementing proactive multidisciplinary consultations and enhancing communication between surrogates and providers. While these strategies are crucial for ensuring informed decision-making and increasing surrogate satisfaction, they do not directly address surrogates’ internal capacity to manage their psychological experiences, which is essential when making high-stakes decisions amidst profound uncertainty and maintaining long-term psychological well-being [5,16].

Consequently, ICU investigators have piloted emotionally oriented interventions grounded in principles of clinical psychology. These include 2 clinician-led interventions delivered in face-to-face or telehealth formats, and a third that involves implementing a commercially available mental health app (Sanvello) [17-20]. This small body of evidence supports the feasibility, acceptability, and preliminary efficacy of these interventions. However, only 1 intervention has undergone a fully powered clinical trial, which revealed no significant between-group effects on the primary psychological outcomes (eg, anxiety, depression, and posttraumatic stress symptoms) [19,21]. Furthermore, several limitations persist. Clinician-led interventions may be too resource-intensive to scale in less-equipped health care settings. Commercial applications like Sanvello offer scalability but lack customization for surrogates’ specific psychological needs. Most importantly, these interventions primarily address long-term psychological symptoms post-ICU discharge, offering surrogates little guidance when managing acute distress during the early days of the patient’s ICU stay, a critical period when decisions can significantly influence patients’ clinical trajectories and necessitate even more complex decisions from surrogates [22].

Therefore, we developed the Reappraisal-Enhanced Foundation for Regulating Affect and Managing Emotions (REFRAME) intervention, a tablet-based app designed to help surrogates manage acute psychological distress during the critical early phase of a patient’s ICU stay. Grounded in the Process Model of Emotion Regulation, REFRAME both educates surrogates and promotes the use of cognitive reappraisal (reappraisal) for managing psychological distress during the patient’s ICU stay [23-27]. Reappraisal is widely regarded as a practical, adaptable, and highly effective strategy that surrogates can use to reframe negative stressors and reduce the intensity of ensuing psychological distress [28-30]. For instance, a surrogate can use reappraisal to alleviate the emotional burden of withdrawing life-sustaining care for the patient by reframing their role as an opportunity to honor the patient’s preferences. We found that surrogates with stronger reappraisal tendencies experience less intense symptoms of psychological distress and use fewer cognitive resources when receiving both active and passive forms of decision support [31,32].

In summary, considering the urgent need for on-demand interventions that assist surrogates in managing acute psychological distress, their openness to emotionally focused interventions offered in digital formats, the compelling evidence showing the feasibility of such interventions in the ICU setting, and the extensive research highlighting the effectiveness of reappraisal across various cultures and contexts, we sought to evaluate the implementation and preliminary efficacy of REFRAME in a prospective sample of ICU surrogates. We hypothesized that REFRAME would be feasible to implement, perceived as acceptable and suitable by surrogates, and linked to a more significant reduction in psychological distress symptoms compared to standard ICU care. To test these hypotheses, we pursued the following aims: (1) evaluate the implementation (ie, feasibility, acceptability, and appropriateness) of REFRAME and (2) examine the preliminary effects of REFRAME on symptoms of psychological distress (ie, anxiety and depression).

## Methods

Our reporting practices for this study were guided by the CONSORT (Consolidated Standards of Reporting Trials) statement for nonrandomized pilot and feasibility studies [33,34].

### Ethical Considerations

Before conducting screening, recruitment, and data collection procedures, we obtained approval from the Institutional Review Board (IRB) at University Hospitals Cleveland Medical Center (IRB00001691), in accordance with the ethical standards of the Declaration of Helsinki. Participants provided written informed consent before data collection, understanding that all study data would be deidentified and stored securely in an encrypted REDCap (Research Electronic Data Capture; Vanderbilt University) database prior to analysis. We offered a US $25 gift card as compensation for participants who completed all study procedures.

### Design

Initially, we planned to conduct this study as a parallel group randomized trial; however, we encountered usability issues with the REFRAME intervention that prevented us from implementing it at the start of our recruitment period. Therefore, we modified our design to a pilot before-and-after (nonrandomized) study with a 1:1 group allocation ratio. We enrolled the first 20 participants in our control condition (usual care [UC]), followed by participants in the intervention condition (REFRAME). We oversampled the intervention group, aiming to complete all 3 intervention modules with 20 participants.

### Control Condition (UC)

We defined UC as the communication and decisional support routinely provided to surrogate decision-makers of ICU patients. UC practices varied among clinical teams, ranging from brief interactions to extensive discussions. We recognize that research procedures, such as obtaining informed consent, may have heightened participants’ awareness of their emotional needs, but these processes were not considered a structured intervention.

### Intervention Condition (REFRAME)

REFRAME is a tablet-based app designed to support emotion regulation in ICU surrogates. The intervention consisted of 3 sequential modules corresponding to stages in the Extended Process Model of Emotion Regulation, with the user interface design grounded in Cognitive Load Theory [24,35]. Apart from incorporated assessment components (eg, surveys), all educational and instructional components were audio guided with corresponding closed captioning. For minor disruptions (<15 minutes) or attentional lapses, participants were allowed to repeat previous content from their desired location within the module. We required participants to restart the intended module after major interruptions (>15 minutes). Each module required 10-15 minutes to complete. To minimize the risk of contamination and minimize staff burden, bedside clinicians were not involved in delivering or reinforcing REFRAME and received no training in intervention principles.

We designed module 1 to evaluate the users’ beliefs about emotion regulation [36], establish a foundational knowledge base related to participants’ awareness of physiological and psychological symptoms of anxiety and depression, introduce the concept of reappraisal, and provide an example of how to apply it within the context of the surrogate role (Figure S1 in Multimedia Appendix 1). Module 2 further expands upon this knowledge by introducing users to different reappraisal strategies (ie, reconstruing and repurposing) with context-relevant examples (Figures S2 and S3 in Multimedia Appendix 1), guiding participants through an evaluation of the various applied examples, and establishing an implementation intention to use their preferred strategies when they become aware of symptoms discussed in module 1 [27,37,38]. Finally, module 3 integrates the principles of reappraisal to evaluate a hypothetical tracheostomy and feeding tube decision-making scenario from multiple perspectives: self-interested, projected interests, best interest, and substituted interest (Figure S4 in Multimedia Appendix 1) [39].

### Participants

We enrolled adults (aged ≥18 years) recognized by the ICU team as the legally authorized representative for a decisionally incapacitated adult who had been residing in the ICU for at least 48 hours and was not expected to be discharged in the following 24 hours. We excluded participants who could not read or speak English because the study instruments and the study intervention were available only in English. We recruited all participants from the cardiothoracic, medical, neurological, and surgical ICUs at a tertiary medical center in northeast Ohio.

### Sample Size Justification

We determined the sample size for this pilot trial using recommendations for estimating variance and minimizing overall trial size in pilot and main trials. For small- to medium-effect sizes (δ=0.2-0.5), Whitehead et al [40] recommend a pilot trial sample size of 20 participants per arm to estimate variance with reasonable precision while avoiding unnecessary participant recruitment. Similarly, a large group of investigators recently reported small- to medium-effect sizes (Cohen *d*=0.24-0.39) of a cognitive reappraisal intervention on negative emotions across a multinational sample (N=21,644) [30]. Thus, we sought to recruit 20 participants per study arm to evaluate REFRAME’s implementation.

### Instruments

#### Implementation Outcomes (Aim 1)

#### Feasibility

We measured feasibility—the extent to which an intervention can be successfully implemented—by evaluating the completion percentages of participants assigned to the intervention condition. We also monitored participants’ adherence to the intervention protocol during delivery and reasons for missed doses. This approach allowed us to gauge the intervention’s practicality and identify potential implementation barriers for future iterations [41]. We defined completion as the user reaching the end of the module section containing postmodule feedback assessments. Using previous recommendations and findings, we sought an a priori benchmark of 70% completion across all 3 modules as supportive feasibility evidence for REFRAME’s implementation [41-43].

#### Acceptability and Appropriateness

We measured acceptability—the evaluation of one’s satisfaction with an intervention—with the Acceptability of Intervention Measure (AIM); we measured appropriateness—the assessment of an intervention’s compatibility within a given context—with the Intervention Appropriateness Measure (IAM). Both scales contain 4 items scored on a 5-point Likert scale. A total score is calculated by averaging the item responses, with higher scores indicating greater acceptability or appropriateness. Before analyzing the study data, we defined mean item scores of 4 or higher on the 5-point scale as evidence of acceptability and appropriateness, consistent with the “agree” response option for these measures. The scales’ creators provide extensive evidence of the AIM’s and IAM’s readability, structural validity, construct validity, reliability, and sensitivity to change [44]. Across multiple administrations of each measure, the internal consistencies (Cronbach α) for AIM and IAM ranged from 0.82-0.97 and 0.92-0.97, respectively. In addition, after each module, participants were asked to share what they liked and what could be improved about REFRAME, which they responded to directly in the app with a standard keyboard (ie, “QWERTY”).

#### Psychological Distress (Aim 2)

We measured symptoms of psychological distress with the PROMIS (Patient Reported Outcomes Measurement System) Emotional Distress–Anxiety and Depression short forms. Both scales contain 4 items scored on a 5-point Likert scale. We calculated the sum of item responses for each scale to calculate raw scores, which we then converted to T scores using precalculated conversion tables [45]. Higher scores indicate greater severity of psychological distress. Previous investigators have provided robust evidence of the Anxiety and Depression short forms’ structural and construct validity, as well as reliability [46,47]. At baseline in our sample, the internal consistency (Cronbach α) of the Anxiety and Depression short forms was 0.87 and 0.89, respectively.

### Sociodemographic Characteristics

We used recommended items from the Phenotypes and eXposures Social Determinants of Health Toolkit to measure the surrogate’s age (years), gender identity, racial identity, marital status, household income, education, and employment status; we also used previous team-generated items to measure the surrogate decision-maker’s relationship to the patient, advance care planning experience, and decision-making experience [48].

### Perceived Stress

We measured surrogates’ baseline perceived stress levels with the 10-item Perceived Stress Scale (PSS) [49]. Participants rated each item on a 5-point Likert scale ranging from “never” (0) to “very often” (4). We calculated total scores by summing item responses, with higher scores reflecting greater perceived stress. To align the PSS timeframe with the surrogate’s acute ICU experience and other study instruments, we modified its reflection period from “over the past month” to “in the last week.” This modification is supported by evidence of the PSS’s psychometric robustness over shorter timeframes [50], its strong correlations with baseline anxiety (*r*=0.68; *P*<.001) and depressive symptom severity (*r*=0.66; *P*<.001), and its excellent internal consistency (α=0.90) in our sample.

### Participant Screening and Recruitment

We recruited participants from the adult cardiothoracic, medical, neuroscience, and surgical ICUs at University Hospitals Cleveland Medical Center in Cleveland, Ohio. We followed a standardized, IRB-approved protocol to ensure consistency. Each day, a research assistant (RA) would screen the electronic medical records of adult ICU patients to verify their age (≥18 years) and ICU length of stay (≥48 hours). For these patients, the RA would confer with the patient’s bedside provider (ie, nurse, nurse practitioner, physician, or physician assistant) to confirm discharge status (≥24 hours) and evaluate the patient’s decision-making capacity using an IRB-designated assessment: (1) Is the patient alert and able to communicate? (2) Is the patient comfortable enough to communicate? and (3) Is the patient medically stable enough for an informed consent discussion? The patient was deemed incapacitated if any of the assessment parameters were not met. In rare cases of uncertainty from the provider or RA, the RA would consult the principal investigator (GP) for a designation. Upon confirming the patient criteria, the RA would consult the provider to identify and introduce the patient’s surrogate. Then, the RA would confirm the surrogate’s age (≥18 years), verify their ability to read and understand English, introduce the study, and ascertain their interest in participating. For interested surrogates, the RA would initiate an informed consent discussion within the patient’s room or a preferred location near the ICU.

### Data Collection

The RA would confirm and notify the surrogate of their group allocation upon obtaining written informed consent. Next, they would administer the sociodemographic questionnaire, the psychological distress instruments, and other instruments not referenced in this study. This interview (T0) required about 20 minutes. The RA conducted 3 follow-up interviews (T1-T3) 24-48 hours after each preceding interview. For those in the UC group, the RA would administer the psychological distress instruments in person or by telephone; these interviews lasted 5-10 minutes. For those in the REFRAME condition, the psychological distress, acceptability, and appropriateness assessments were embedded within the interventional modules, with each module requiring 10-15 minutes for completion. The REFRAME modules were not scheduled relative to specific clinical milestones. Because this was a feasibility pilot, our priority was to evaluate implementation procedures and maintain comparable assessment timepoints between study arms, rather than synchronizing intervention delivery with specific clinical milestones.

### Treatment Fidelity

We incorporated fidelity-enhancing strategies across 4 of the 5 domains outlined by the National Institutes of Health Behavior Change Consortium framework [51,52]. We did not measure the enactment of treatment-related skills because the primary focus of this study was on evaluating REFRAME’s implementation in surrogates.

- Study Design: We operationalized REFRAME using the Standard and Extended Process Model of Emotion Regulation [20,21]. We developed detailed guides to designate module content, sequence, design, and user interface, ensuring alignment with the theoretical framework.
- Training: To ensure consistent intervention delivery, we provided standardized training for research staff, including role-plays, booster sessions, and random delivery audits.
- Delivery: The modular content was identical for all recipients, and we followed prespecified protocols for reviewing content following short interruptions (≤15 minutes) and restarting the module following lengthy interruptions (>15 minutes).
- Receipt: All educational content was delivered via prerecorded audio with synchronized subtitles and accompanying visual displays to promote intervention receipt. Reviews of key modular content were embedded at the end of each module and the beginning of the next module.

### Analysis

Before our primary analyses, we examined descriptive statistics to assess variable distributions, identify potential outliers and influential cases, and evaluate assumptions for our quantitative analyses. We assessed patterns of missing data with descriptive summaries, Pearson chi-square tests, independent-sample *t* tests (2-tailed), and logistic regression.

#### Implementation Outcomes (Aim 1)

We conducted all quantitative analyses using SPSS (version 29; IBM Corp). To evaluate the feasibility, acceptability, and appropriateness of REFRAME, we analyzed module completion percentages and raw scores on the AIM and IAM using descriptive statistics (ie, means, SDs, and frequencies). To contextualize our quantitative findings, we examined reasons for missed doses and conducted a thematic analysis of participants’ typewritten feedback, collected after each module. These open-ended responses typically ranged from 1 to 3 sentences. A total of 2 independent coders used open coding to manually categorize responses, identifying different barriers and facilitators to REFRAME’s implementation. They resolved discrepancies through discussion until reaching a consensus. We selected thematic analysis because it allowed us to group common patterns from participant feedback while remaining flexible to unanticipated insights related to REFRAME’s implementation [53].

#### Psychological Distress (Aim 2)

Following methodological recommendations for early-phase behavioral intervention studies [34,54,55], we examined changes in psychological distress severity from baseline (T0) to 1 week postbaseline (T3) between the intervention (REFRAME) and UC groups by calculating individual change scores (Δ=T3–T0) for anxiety and depressive symptoms and comparing mean changes between groups with independent samples *t* tests. We quantified between-group effects by interpreting Cohen *d* and corresponding 95% CIs, using standard conventions for small (≥0.2), medium (≥0.5), and large (≥0.8) effects [56].

Given our sequential, nonrandomized design, we conducted post hoc sensitivity analyses using linear mixed-effects models to evaluate the robustness of our findings. In each model, we included fixed effects for group (UC=0; REFRAME=1), timepoint (T0, baseline=0; T3, 1 week postbaseline=1), and their interaction (group × timepoint). Although this analysis was underpowered for multivariable inference, we added gender (man=0; woman=1), patient relationship (spouse/domestic partner=0; nonspouse/nondomestic partner=1), previous health care decision-making experience (no=0; yes=1), and perceived stress as a priori covariates to reduce residual variance and ensure that between-group differences were not confounded by known correlates of psychological distress in surrogates [2]. We specified random intercepts for each participant to account for baseline differences in psychological distress severity and used a diagonal covariance structure to model the repeated measurements of outcomes. The model coefficients were estimated using restricted maximum likelihood with Satterthwaite-adjusted degrees of freedom.

## Results

### Participant Enrollment and Attrition

Between August 2022 and June 2023, we screened 2587 patients for eligibility, with the majority being ineligible due to a pending discharge status (989/2587, 38.2%), an ICU length of stay of less than 48 hours (583/2587, 22.5%), or possessing capacity for decision-making (387/2587, 15.0%). Of the 95 eligible surrogates (3.7%), a total of 46 (48.4%) declined participation. During the 10-month recruitment period, we enrolled 49 surrogates (4.8 per month). The first 20 participants were allocated to UC, and the following 29 to REFRAME. Notably, 1 participant assigned to the REFRAME condition withdrew from the study after providing informed consent but before baseline data collection, resulting in a final analytic sample of 48 surrogates.

**Figure 1:**
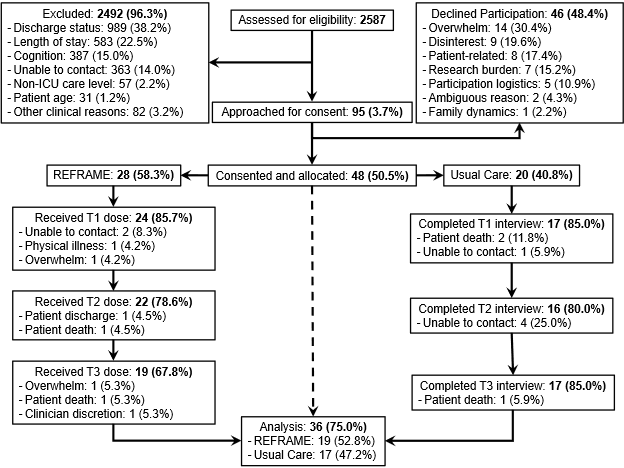
CONSORT (Consolidated Standards of Reporting Trials) flowchart of participant enrollment, allocation, and adherence. ICU: intensive care unit.

As shown in Figure 1, we experienced greater attrition in the REFRAME group (n=9) than in the UC group (n=3), with withdrawal reasons including patient discharge or death and the participant either being nonresponsive, feeling overwhelmed, or physically ill. Despite this, study group (*χ*^2^_1_=1.8; *P*=.18) and all other proposed covariates were not associated with study attrition, suggesting our data were at least missing at random.

### Participant Baseline Characteristics

Table 1 compares surrogate baseline characteristics by study group. Overall, our sample’s average age was 58.6 (SD 13.2) years, and it was predominantly comprised of women (36/48, 75%) and White individuals (41/48, 85.4%). Most participants were married (33/48, 68.8%), employed full-time or part-time (27/48, 56.2%), nonbaccalaureate educated (26/48, 54.2%), and living in a household with an annual income of less than US $100,000 (25/48, 52.1%). Most surrogates had not discussed the patient’s preferences regarding cardiopulmonary resuscitation (32/48, 66.7%), mechanical ventilation (30/48, 62.5%), artificial nutrition (40/48, 83.3%), or dialysis (33/48, 68.8%), despite more than half reporting that they had discussed end-of-life care (25/48, 52.1%). More than 62.5% (30/48) of surrogates reported moderate to severe anxiety symptoms, and one-third (16/48, 33.3%) endorsed moderate to severe depressive symptoms. While the baseline characteristics of participants were mostly balanced across groups, 75% (15/20) of surrogates in the UC group were the patient’s spouse, compared with 39% (11/28) in the REFRAME group (Pearson *χ*^2^_1,48_=6.0; *P*=.01). Furthermore, 95% (19/20) of surrogates in the UC group lacked experience in making health care decisions for the patient, a larger proportion than surrogates in the REFRAME group (19/28, 67.9%) (Fisher exact test, *P*=.03).

**Table 1:** Baseline characteristics of enrolled surrogates by study group (N=48). Data available for 26 participants in the Reappraisal-Enhanced Foundation for Regulating Affect and Managing Emotions (REFRAME) group and 17 participants in the usual care (UC) group due to declined responses.

Variable			REFRAME (n=28)		UC (n=20)		*P* value^a^	
Age (years), mean (SD)			58.1 (13.8)		59.2 (12.6)		.77	
**Gender, n (%)**								.499
	Woman	20 (71.4)		16 (80)				
	Man	8 (28.6)		4 (20)				
**Racial identity, n (%)**								.37
	White	25 (89.3)		16 (80)				
	Non-White	3 (10.7)		4 (20)				
**Marital status, n (%)**								.43
	Married	18 (54.5)		15 (75)				
	Not married	10 (35.7)		5 (25)				
**Relationship to patient, n (%)**								.01
	Spouse/domestic partner	11 (39.3)		15 (75)				
	Nonspouse/nondomestic partner	17 (60.7)		5 (25)				
**Employment status, n (%)**								.66
	Employed (full/part time)	15 (53.6)		12 (60)				
	Not employed	13 (46.4)		8 (40)				
**Highest education attained, n (%)**								.96
	≤High school	6 (21.4)		5 (25)				
	Some college/associate degree	9 (32.1)		6 (30)				
	≥Bachelor’s degree	13 (46.4)		9 (45)				
**Household income (US $), n (%)**								.49
	<60,000	9 (34.6)		5 (29.4)				
	60,000-99,999	8 (30.8)		3 (72.7)				
	≥100,000	9 (34.6)		9 (52.9)				
**Prior ACP^b^ conversations (Yes), n (%)**								
	CPR^c^	10 (35.7)		6 (37.5)		.68		
	Mechanical ventilation	10 (35.7)		8 (40)		.76		
	Artificial nutrition	5 (17.9)		3 (37.5)		.56		
	Dialysis	7 (25)		8 (40)		.27		
	End-of-life care	14 (50)		11 (55)		.73		
**Decision-making experience, n (%)**								.03
	No	19 (67.9)		19 (95)				
	Yes	9 (32.1)		1 (5)				
Perceived stress raw score, mean (SD)			24.2 (4.4)		21.8 (4.2)		.06	

^a^*P* values were calculated using Pearson chi-square or Fisher exact tests for categorical variables and independent-samples *t* tests (2-tailed) for continuous variables.

^b^ACP: advance care planning.

^c^CPR: cardiopulmonary resuscitation.

### Implementation Outcomes (Aim 1)

#### Feasibility

Of the 28 enrolled surrogates in the REFRAME group with baseline data, a total of 19 received all 3 intended intervention doses (19/28, 67.9%). Of those (n=9) who did not receive all intended doses, a total of 3 (33.3%) missed doses because of patient-related factors (eg, discharged from ICU or patient death), 3 (33.3.%) claimed they were too overwhelmed or busy to remain committed to the study protocol, 2 (22.2%) were not responsive to our follow-up efforts, and 1 (11.1%) participant developed an illness that prevented them from visiting the ICU.

#### Acceptability and Appropriateness

##### Quantitative Findings

Surrogate perceptions of REFRAME’s acceptability and appropriateness were positive across all 3 modules (Table 1). Over 70% of surrogates’ average scores were at least 4.00 across all 3 modules, indicating that the intervention was acceptable and appropriate. For acceptability, we observed favorable ratings (≥4.00) for 79.2% (19/24), 78.3% (18/23), and 73.7% (14/19) of participants for each module. Appropriateness followed a similar trend, with 70.8% (17/24), 73.9% (17/23), and 78.9% (15/19) of participants providing favorable ratings for the same modules (Table 2).

**Table 2:** Acceptability and appropriateness summary statistics by Reappraisal-Enhanced Foundation for Regulating Affect and Managing Emotions (REFRAME) module.

Module (timepoint)	Frequency, n	Acceptability			Appropriateness	
		Minimum-maximum	Mean (SD)	Minimum-maximum		Mean (SD)
1 (T1)^a^	24	2.75-5.00	4.36 (0.70)	3.00-5.00		4.34 (0.68)
2 (T2)^b^	23	3.00-5.00	4.37 (0.69)	3.25-5.00		4.35 (0.66)
3 (T3)^c^	19	1.00-5.00	4.28 (1.00)	1.25-5.00		4.26 (1.00)

^a^T1: 1-2 days post enrollment.

^b^T2: 3-4 days post enrollment.

^c^T3: 5-6 days post enrollment.

##### Qualitative Findings

To provide deeper insight into our quantitative findings, we identified themes related to strengths and areas for improvement among participants across the three intervention modules. Table S1 in Multimedia Appendix 2 summarizes the primary themes and subthemes we identified in our analysis with supporting participant quotes. Across the modules, participants appreciated the intervention’s ability to validate their emotional experiences, introduce practical emotion regulation skills, and provide an example of how they can frame decisions they may encounter during the patient’s ICU stay. Importantly, participants shared that we could further improve the intervention by tailoring its content to their unique experiences, enhancing its usability, and considering how to adeptly present emotionally charged content.

##### Module 1: Introducing Emotion Regulation

Surrogates highlighted several ways the first module validated their emotional experiences, demonstrated the importance of practical tools for managing those emotions, and emphasized the necessity for adjustable narration pacing. A common theme among participants was the appreciation for normalizing their feelings. For instance, one participant noted, “It is helpful to point out that stress and sadness are normal and that there are ways to deal with it,” while another wrote, “It helps me focus on my feelings and self-care while also focusing on my loved one.” Furthermore, participants recognized the value of providing a practical framework for managing emotions, such as refocusing strategies during distress. One participant remarked, “It gives family members a practical tool to manage emotions.” In addition to these insights, participants provided feedback on enhancing usability by allowing control over narration pacing. One participant explained, “I would benefit from reading through the entire presentation at my own reading speed.”

##### Module 2: Reappraisal Strategies

Surrogates provided positive feedback on the second module, highlighting the introduction of reappraisal techniques and the reinforcement of existing coping mechanisms that were immediately applicable. One participant shared, “This seems to be exactly what I need. I have tried several strategies, and they have helped me see a better way to cope.” Users appreciated how the module reinforced existing coping mechanisms while introducing varied approaches. As another participant noted, “This is a great reminder for me to use a familiar process in times of stress.” However, some participants expressed concerns about the content’s simplicity and repetitiveness, especially for those who may be more adept at managing stress. One remarked, “It seems a bit too simplistic because you can guess what point is going to be made next.” Thus, while the module was well received for its practical approaches, there remains room for greater depth and engagement.

##### Module 3: Decision-Making Application

Surrogates expressed strong appreciation for the third module, noting its importance in providing relevant tools for navigating real-life decisions in the ICU. Participants highlighted the module’s practicality in real-life situations, with one stating, “The scenarios were appropriate for decisions I have had to make for my loved one.” Another participant remarked on its support for making “clear and difficult decisions” during emotionally charged moments. However, one participant raised a crucial point regarding the need to consider a surrogate’s emotional readiness when addressing challenges such as end-of-life decisions: “The death discussion is too scary for that time when the family member is in the ICU.”

### Effects on Psychological Distress (Aim 2)

Table 3 displays within- and between-group changes in psychological distress severity from baseline (T0) to approximately 1 week postbaseline (T3). At T0, anxiety and depressive symptoms were higher in the REFRAME group (*t*_34_=−1.69; *P*=.10 and *t*_34_=−1.33; *P*=.19), and at T3, psychological distress severity decreased in both groups. However, anxiety symptoms decreased by an average of 2.2 points more in the REFRAME group (95% CI −2.08 to 6.53), and depressive symptoms decreased by an average of 4.01 points more in the REFRAME group (95% CI −0.01 to 8.02). These differences favoring the REFRAME group correspond to a small effect for anxiety symptoms (*d*=0.35, 95% CI −0.31 to 1.01) and a medium effect for depressive symptoms (*d*=0.68, 95% CI −0.001 to 1.35).

**Table 3:** Within- and between-group changes in psychological distress severity from baseline (T0) to 1 week postbaseline (T3) in surrogate decision-makers.

Variable and group		T0^a^, mean (SD)	T3^b^, mean (SD)	Δ mean (SD)^c^	Between-group Δ (95% CI)^d^		Cohen *d* (95% CI)^e^	
**Anxiety**						2.22 (−2.08 to 6.53)		0.35 (−0.31 to 1.01)
	UC^f^	59.78 (10.61)	56.35 (10.04)	3.43 (6.37)				
	REFRAME^g^	65.21 (8.67)	59.56 (9.81)	5.65 (6.33)				
**Depression**						4.01 (−0.01 to 4.08)		0.68 (−0.001 to 1.35)
	UC	52.71 (8.04)	52.20 (8.17)	0.51 (3.95)				
	REFRAME	56.97 (10.77)	52.45 (10.18)	4.52 (7.23)				

^a^T0: baseline.

^b^T3: 5-6 days postbaseline.

^c^Change from T0 to T3 (Δ=T₃–T₀).

^d^Between-group difference in mean change scores (REFRAME–UC).

^e^Standardized mean difference; small ≥0.2, medium ≥0.5, and large ≥0.8.

^f^REFRAME: Reappraisal-Enhanced Foundation for Regulating Affect and Managing Emotions.

^g^UC: usual care.

### Sensitivity Analysis

**Figure 2:**
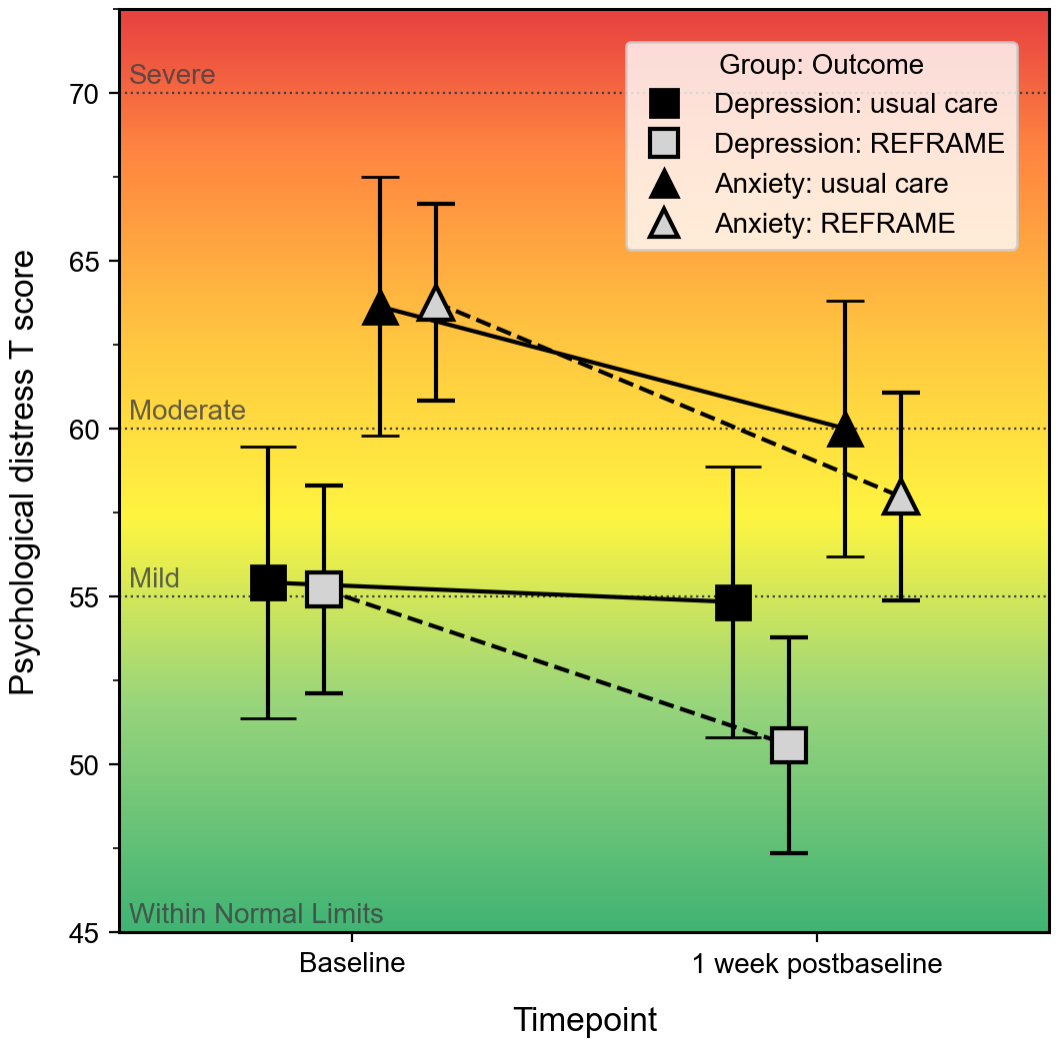
Estimated marginal means (EMMs) of anxiety and depressive symptom severity (PROMIS [Patient Reported Outcomes Measurement Information System] T scores) from baseline (T0) to approximately 1 week postbaseline (T3) by study group. Lines and points represent model-estimated EMMs from mixed-effects models that adjust for gender, patient relation, prior decision-making experience, and perceived stress; error bars indicate 95% CIs. Usual care is represented with black-filled symbols connected by solid lines, and Reappraisal-Enhanced Foundation for Regulating Affect and Managing Emotions (REFRAME) with gray-filled symbols connected by dashed lines. Anxiety severity is indicated by triangles, and depression severity is indicated by squares. The background color bands illustrate PROMIS severity categories for interpretation: green indicates within normal limits (<55), yellow represents mild (55-59.9), orange denotes moderate (60-69.9), and red indicates severe (≥70).

The findings from our sensitivity analysis align with those from our primary analysis (Tables S2 and S3 in Multimedia Appendix 2). Anxiety symptoms decreased in both groups from T0 to T3, with the REFRAME group reporting a larger decrease (b=−2.14, 95% CI −6.20 to 1.92). Of our prespecified covariates, perceived stress (b=1.38, 95% CI 0.99-1.78), identifying as a woman (b=−4.82, 95% CI −8.74 to −0.89), and having prior decision-making experience (b=−6.26, 95% CI −11.17 to −1.35) were positively associated with anxiety severity. Similarly, depressive symptoms decreased in both groups from T0 to T3, with the REFRAME group reporting a larger decrease (b=−4.06, 95% CI −7.91 to −0.21). Unlike the anxiety model, perceived stress was the only covariate associated with depression severity at *P*<.05 (b=1.36, 95% CI 0.93-1.79). Figure 2 depicts the model-estimated marginal means for changes in anxiety and depressive symptoms from T0 to T3, adjusted for all prespecified covariates, providing convergent evidence for the robustness of the observed patterns in our primary analysis.

## Discussion

### Implementation Outcomes (Aim 1)

#### Principal Findings

We present evidence that REFRAME, our tablet-based emotion regulation intervention, can be feasibly implemented, is user-friendly, and is relevant to surrogate decision-makers of patients with critical illness. Over a 10-month recruitment period, we enrolled and allocated 48 out of 95 eligible surrogates (50.5%). Two-thirds of participants in the intervention group completed all 3 modules, with over 70% rating the intervention as acceptable and appropriate. Emerging themes from our qualitative findings indicate that surrogates valued the intervention’s ability to normalize their emotional experiences, offer practical reappraisal techniques for managing distressing emotions, and incorporate reappraisal principles into their decision-making. However, participants also identified several areas for improvement, including adjusting the intervention’s pacing, addressing emotionally charged content with greater sensitivity, and tailoring the content to individual needs. Compared with the existing literature, our findings highlight the potential of REFRAME to provide crucial support to this particularly vulnerable population.

#### Comparison With Prior Work

The feasibility of enrolling and retaining ICU surrogates in clinical trials is often hindered by their concerns for the patient or limited emotional bandwidth. Many patient-oriented trials fail to reach their intended sample size, with refusal rates approaching 70% [57-60]. However, investigators conducting surrogate-oriented trials of similar psychological interventions have reported higher enrollment rates, even though, before REFRAME, these interventions had not been directly implemented during ICU stays. For example, investigators piloting a telephone-based coping skills training (CST) intervention for acute lung injury survivors and their informal caregivers recruited 10 dyads across 4 sites between 2009 and 2010 (~0.8 dyads/month), achieving 92% session completion and unanimous endorsement of the intervention’s usefulness [19]. Similarly, investigators piloting Enhancing and Mobilizing the Potential for Wellness and Resilience (EMPOWER)—a clinician-led mental health intervention delivered in face-to-face and telehealth formats—enrolled approximately 2 participants/month, with low refusal rates (23%) and high adherence rates (89%), but moderate attrition after 3 months (37%) [18]. In comparison, we enrolled nearly 5 participants per month, exceeding the per-site pace reported in the CST and EMPOWER trials, while achieving a slightly lower retention rate (~68%) in the REFRAME condition, which approached our a priori goal of 70% retention. This is expected, as we sought to recruit surrogates during a distressing and unpredictable period of the patient’s ICU stay, and differences in population acuity and timing (during vs after ICU) limit direct comparisons of enrollment and retention across studies.

While interventions like CST and EMPOWER are promising, they may require significant resources, which can limit their scalability across different ICU contexts. Thus, we deliberately chose a tablet-based platform for REFRAME to provide on-demand and scalable psychological support. For example, investigators trialing the web-based decision aid, Electronic Collaborative Decision Support (eCODES), reported high acceptability ratings, with enrollment, adherence, and attrition percentages comparable to the EMPOWER trial [61,62]. Likewise, in 2 pilot trials of the commercially available Sanvello mental health app, investigators reported moderate-to-high satisfaction and 20% attrition over 60 days, though engagement varied substantially [17,20]. Notably, EMPOWER, eCODES, and Sanvello targeted psychological outcomes weeks to months after ICU admission.

In contrast, we designed REFRAME to target psychological distress during the acute phase of critical illness—when well-intentioned routine procedures and urgent interventions can escalate into highly complex clinical scenarios—demanding surrogates to make life-altering decisions in the face of intense uncertainty [22]. In this period, surrogates are often overwhelmed at this early stage with balancing new and unfamiliar information, contemplating high-stakes decisions, and managing acute psychological distress. Considering this context may partly explain why we had lower enrollment and adherence percentages than interventions delivered later in the ICU stay or post discharge. Consistent with previous findings, many eligible surrogates felt too overwhelmed to enroll in this study [63,64]. Nonetheless, supporting surrogates during this critical period may ultimately enhance value-concordant care for patients while mitigating long-term psychological morbidity for surrogates.

### Effects on Psychological Distress (Aim 2)

#### Principal Findings

Both anxiety and depressive symptoms decreased from moderate to mild severity across study groups from baseline (T0) to approximately 1 week postbaseline (T3). However, anxiety improvement was not linked to group assignment. In contrast, depressive symptoms in the REFRAME group declined from mild severity to near-normal limits, while those in the UC group maintained mild depressive severity throughout the study period. The magnitude of this change met the PROMIS Depression minimally important difference (≈3-4 T score points) threshold established from 3 separate randomized trials (N=651), potentially indicating a clinically significant effect [65]. However, because participants in the intervention group entered the study with slightly higher baseline distress and distress naturally subsides over the first few days of an ICU stay, the observed improvement may reflect circumstantial adaptation or regression to the mean [3,66].

Nonetheless, our primary analysis findings are further supported by our more analytically robust sensitivity analysis, in which perceived stress emerged as the strongest predictor across all variables in both models. Although women, patient spouses or partners, and individuals with prior health care decision-making experience reported higher levels of psychological distress, these associations’ CIs were wide in both models and only excluded zero in our anxiety model. Notably, our limited sample size restricted our ability to detect smaller effects that may be clinically meaningful, especially for anxiety symptoms, emphasizing the need to examine REFRAME’s effectiveness in a more adequately powered trial.

#### Comparison With Prior Work

Research on surrogate psychological distress during ICU stays is scarce, often limited to single-time assessments, with follow-ups occurring weeks to months after discharge [2,12,14]. Our previous descriptive study at the same institution found similar distress trajectories between ICU days 3 and 10 [3]. These studies further align with a Spanish study of 104 surrogates that observed decreased anxiety and depressive symptoms from ICU day 3 to post discharge [67]. However, Bolosi et al [68] found stable anxiety and increasing depressive symptoms among 108 Greek surrogates from ICU days 1 to 7, possibly due to cultural, contextual, or methodological differences. Surrogates’ psychological well-being can fluctuate daily, influenced by structural, interpersonal, and intrapersonal factors [5,16,69]. Therefore, routinely monitoring their distress is crucial for implementing targeted interventions that optimize their psychological capacity for sound decision-making and improve long-term psychological outcomes.

Historically, ICU-based clinical trials have mainly focused on fulfilling the informational needs of surrogates, yet they have had limited impact on their long-term psychological well-being [62,70-72]. In contrast, fostering surrogates’ emotion regulation and coping skills through clinician-led or self-directed interventions may improve their resilience against adverse psychological outcomes following the patient’s ICU stay [17,18,21]. These interventions used cognitive-behavioral, acceptance, and commitment therapy techniques, targeting underlying psychological mechanisms that promote long-term psychological well-being. We build on these efforts with REFRAME, highlighting reappraisal as a practical skill for managing acute psychological distress by reframing immediate stressors in the ICU. Furthermore, although baseline depressive symptoms in our sample were mild on average, even subclinical depression can interfere with the cognitive control and motivational processes that support complex medical decision-making, reducing a surrogate’s ability to override emotionally driven impulses in favor of value-based reasoning [73-75]. As a result, mild depressive symptoms during the ICU stay may impair a surrogate’s capacity to integrate information, tolerate ambiguity, and stay engaged in value-based choices, helping to explain the well-documented connection between early depressive symptoms and long-term psychological morbidity after the ICU stay [76-79].

Acute depressive symptoms decreased more in surrogates exposed to REFRAME compared to those unexposed. This finding is consistent with evidence linking reappraisal training to strengthened prefrontal-amygdala connectivity that reduces negative thought patterns observed in depressive psychopathology (eg, rumination and catastrophizing) [80,81]. However, reappraisal may be less preferable or effective for surrogates during emotionally intense periods [82,83]. At baseline, surrogates assigned to REFRAME primarily reported mild (8/28, 28.6%) or moderate (9/28, 32.1%) depressive severity, a psychological context well suited to reappraisal-based strategies. In contrast, most REFRAME-exposed surrogates reported moderate (12/28, 42.9%) or severe (7/28, 25.0%) anxiety symptoms, which may hinder reappraisal efforts. Surrogates often manage intense anxiety and sleep deprivation, diminishing their capacity for emotion regulation and information processing [3,4,84,85]. In such contexts, alternative strategies such as acceptance or distraction may be less cognitively demanding and offer immediate relief, thereby enhancing future reappraisal efforts [86,87]. Future interventional research should promote context-dependent emotion regulation strategies flexibly, empowering surrogates to meet immediate emotional needs and foster long-term psychological resilience [29].

### Limitations

Our findings should be considered with several limitations in mind. First, the small, homogeneous sample limits how well the results apply to more diverse populations, such as non-White men. Second, the nonexperimental design introduces selection bias because surrogates who declined participation due to distress may have benefited most from REFRAME, and observed improvements could partly result from regression to the mean, as baseline distress was higher in the REFRAME group. Additionally, participants’ awareness of their group assignment might have influenced their decision to consent, potentially inflating acceptability ratings. Third, while REFRAME aimed to reduce acute psychological distress, the short follow-up restricts the ability to assess long-term or delayed effects on decision-making and outcomes. Fourth, higher attrition in the intervention group could indicate scalability challenges in larger REFRAME evaluations. Fifth, we did not set predefined feasibility benchmarks for enrollment or consent rates, which limits formal comparisons across trials; however, the enrollment rate suggests that a future, fully powered randomized trial of REFRAME is feasible. Lastly, reliance on self-report instruments introduces the possibility of social desirability or recall biases, and the PROMIS measures might reflect immediate ICU-related distress rather than chronic psychiatric symptoms, which should be considered when interpreting short-term changes in distress in this context.

### Conclusions

We provide preliminary evidence that REFRAME can be successfully implemented and is acceptable to ICU surrogates during the acute phase of critical illness, with qualitative feedback supporting its value in normalizing their experiences and providing concrete strategies for managing distress. We also observed improvements in depressive symptoms that favored surrogates in the REFRAME group, although the nonrandomized design and small sample size prevent definitive conclusions about the intervention’s effects. Nonetheless, our findings highlight the practicality of delivering brief, on-demand emotional support to surrogates by tablet-based tools during this high-stress period. A larger, randomized trial with longer follow-up is needed to replicate our findings, evaluate its impact on surrogate decision-making, and examine its downstream influences on long-term psychological well-being. With continued refinement and more rigorous testing, interventions like REFRAME have the potential to improve psychological well-being in surrogates during critical moments of a patient’s ICU stay and to inform future strategies for supporting those making high-stakes medical decisions for a loved one with critical illness.

## Appendix

Multimedia Appendix 1 REFRAME modules' screenshots.

Multimedia Appendix 2 Supplementary tables for quantitative and qualitative analyses.

## Abbreviations

AIM: Acceptability of Intervention Measure
CONSORT: Consolidated Standards of Reporting Trials
CST: coping skills training
eCODES: Electronic Collaborative Decision Support
EMPOWER: Enhancing and Mobilizing the Potential for Wellness and Resilience
IAM: Intervention Appropriateness Measure
ICU: intensive care unit
IRB: institutional review board
PROMIS: Patient-Reported Outcomes Measurement Information System
PSS: Perceived Stress Scale
RA: research assistant
REDCap: Research Electronic Data Capture
REFRAME: Reappraisal-Enhanced Foundation for Regulating Affect and Managing Emotions
UC: usual care

## Data Availability

The data sets generated and analyzed during this study are not publicly available due to ethical considerations; however, they are available from the corresponding author upon reasonable request.
