# Supplementary material for: Evaluation of a Tablet-Based Emotion Regulation Intervention for Surrogate Decision-Makers of Patients With Critical Illness: Pilot Nonrandomized Trial
Source: JMIR Form Res. 2026 Jan 19;10:e73769. doi: 10.2196/73769 (PMC12865353; doi:10.2196/73769)
Supplement: Multimedia Appendix 1 [file formative_v10i1e73769_app1.pdf]

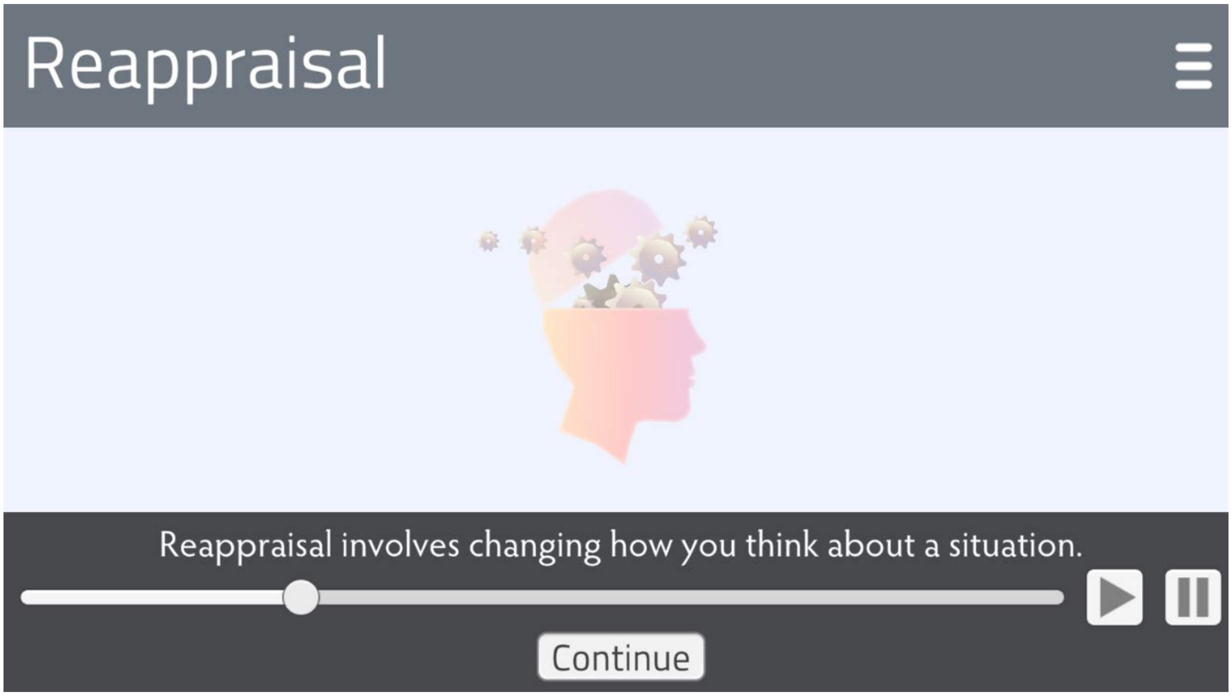

Figure S1. Screenshot from Module 1 of REFRAME, a tablet-based cognitive reappraisal intervention implemented in a nonrandomized trial for surrogate decision-makers of critically ill patients during the first week of an intensive care unit stay (Cleveland, Ohio, 2023). This introductory module presents the concept of cognitive reappraisal as a strategy for managing distress and decision-related stress during the ICU experience.

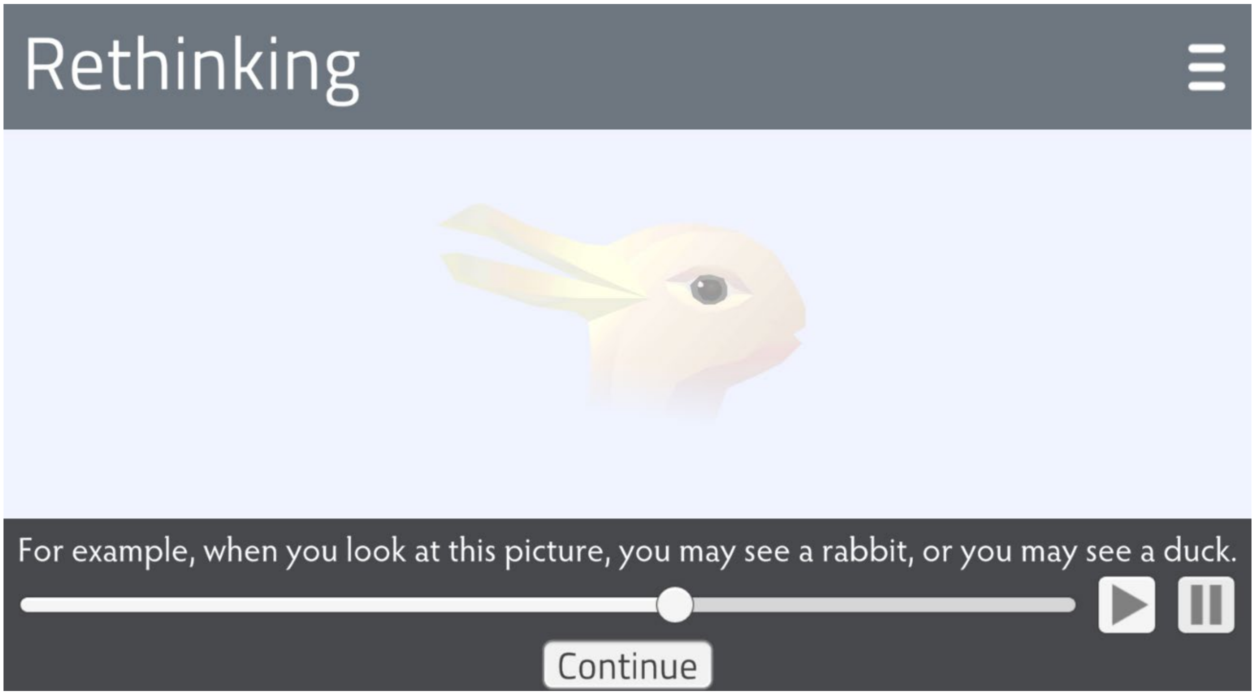

Figure S2. Demonstration of the “rethinking” strategy presented in Module 2 of REFRAME, a tablet-based cognitive reappraisal intervention implemented in a nonrandomized trial for surrogate decision-makers of critically ill patients during the first week of an intensive care unit stay (Cleveland, Ohio, 2023). The ambiguous rabbit–duck image illustrates how shifting one’s interpretation of a situation can alter emotional reactions during stressful medical decision-making.

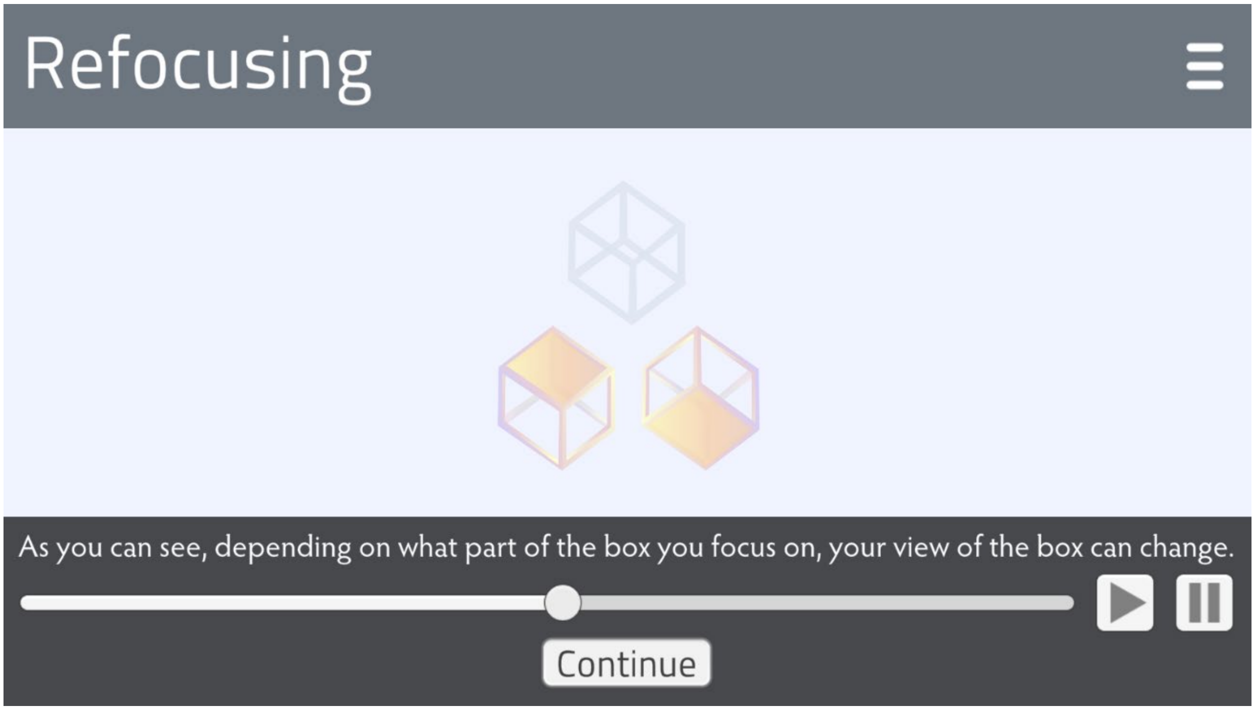

Figure S3. Demonstration of the “refocusing” strategy presented in Module 2 of REFRAME, a tablet-based cognitive reappraisal intervention implemented in a nonrandomized trial for surrogate decision-makers of critically ill patients during the first week of an intensive care unit stay (Cleveland, Ohio, 2023). The cubes appear to change orientation based on the viewer’s focal point, illustrating how redirecting attention to different features of a situation can modulate emotional responses.

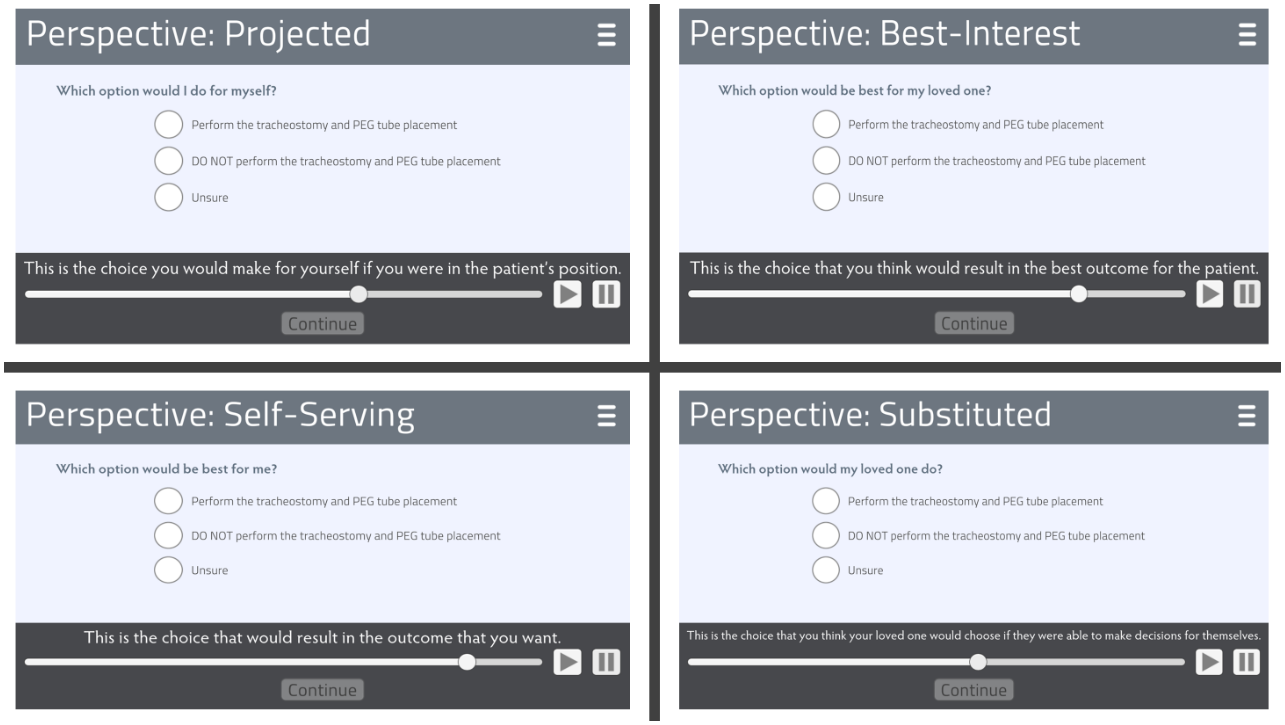

Figure S4. Screenshot from Module 3 of REFRAME, a tablet-based cognitive reappraisal intervention implemented in a nonrandomized trial for surrogate decision-makers of critically ill patients during the first week of an intensive care unit stay (Cleveland, Ohio, 2023). This module guides users to approach decisions from four perspectives (projected, best-interest, self-serving, and substituted judgment) to understand how reappraisal supports value-concordant medical decision-making.

This is a multimedia appendix to a full manuscript published in JMIR Formative Research. For full copyright and citation information, see http://dx.doi.org/10.2196/jmir.73769
