# Supplementary material for: Evaluation of a Tablet-Based Emotion Regulation Intervention for Surrogate Decision-Makers of Patients With Critical Illness: Pilot Nonrandomized Trial
Source: JMIR Form Res. 2026 Jan 19;10:e73769. doi: 10.2196/73769 (PMC12865353; doi:10.2196/73769)
Supplement: Multimedia Appendix 2 [file formative_v10i1e73769_app2.pdf]

Table S1

Participant Qualitative Feedback on the REFRAME Intervention: Themes, Subthemes, and Representative Quotes from Surrogate Decision-Makers of Critically Ill Patients Enrolled in a Nonrandomized Pilot Trial (Cleveland, Ohio, 2023)

Theme	Subtheme	Supporting Quotes
**Module 1 (T1)**[a]		
1. Normalizing Emotional Responses	1A. Recognizing shared experiences	“It is helpful to point out that stress and sadness are normal and there are ways to deal with it.”
	1B. Acknowledging caregiver stress	“I liked that society is also recognizing the stress the family is under and not just the patients.”
2. Facilitating Cognitive Reframing	2A. Reappraisal strategies	“[I liked] the suggestions for rethinking and refocusing.”
	2B. Promoting self-awareness	“[This was] helpful to understand my emotions.”
3. Improving Content Delivery	3A. Adapting pacing	“I would benefit from being able to read through the entire presentation at my own reading speed.”
	3B. Addressing relevance	“This appears to be geared toward people that are being treated and are expected to recover while I am past that point.”
**Module 2 (T2)**[b]		
1. Developing and Reinforcing Accessible Emotion Regulation Skills	1A. Introducing practical approaches	“This seems to be exactly what I need. I have tried several of the strategies given and they have helped me see a better way to cope when I feel like I can't cope any longer.”
	1B. Bolstering existing strategies	“This is a great reminder for me to use a familiar process in times of stress.”
	1C. Simplicity as strength	“Relatively simple approach that a wide variety of audiences can use.”
2. Encouraging Emotional Reflection	2A. Promoting introspection	“I like the different ways of thinking about things.”
	2B. Envisioning the future	“It helped me to understand how I’m going forward.”
3. Improving Content Delivery	3A. Acknowledging differing histories	“Sometimes the older population is already experienced in similar situations and better equipped mentally.”
	3B. Simplicity as weakness	“It seems a bit too simplistic because you can guess what point is going to be made next.”
	3C. Clarifying ambiguity	“A few of the questions are not worded correctly in order to get more accurate responses.”
**Module 3 (T3)**[c]		
1. Empowering Decision-Making	1A. Practical content	“The scenarios were appropriate for decisions I have had to make for my loved one.”
	1B. Real-time reassurance	“[I liked} the support it gave me to make clear and difficult decisions.”
2. Broad Applicability	2A. Other caregiving contexts	“I feel that all people, not just us dealing with very ill loved ones, but just sick can also benefit.”
	2B. In the context of life	“It raises awareness and thoughts about making life-changing events.”
3. Improving Content Delivery	3A. Situational sensitivity	“The death discussion is too scary for that time when the family member is in the ICU.”
	3B. Timing delivery	“Use [this tool] in ICU in the first 3 days of care.”

Table S1
Participant Qualitative Feedback on the REFRAME Intervention: Themes, Subthemes, and Representative Quotes from Surrogate Decision-Makers of Critically Ill Patients Enrolled in a Nonrandomized Pilot Trial (Cleveland, Ohio, 2023)

Theme	Subtheme	Supporting Quotes
	3C. Participant literacy	"It may be helpful to start the first module with a simple survey to reveal the level of medical literacy and have different content based on results."
	3D. Refining interface	"Make the buttons accept a touch better."

[a] 1–2 days post-baseline.
[b] 3–4 days post-baseline.
[c] 5–6 days post-baseline.

Table S2. Summary of fixed and random effects in linear mixed models for predictors of psychological distress severity in a nonrandomized trial of REFRAME, a tablet-based emotion regulation intervention for surrogate decision makers of critically ill patients (Cleveland, Ohio, 2023) (N = 48)

Effect	Estimate	*SE*	*t*	p	95% CI	
					LL	UL
Fixed Effects			Anxiety Symptoms			
Intercept	30.18	5.13	5.88	<.001	19.85	40.52
Study Group (0)[a]	2.03	2.18	0.93	.357	-2.38	6.45
Timepoint (0)[b]	5.77	1.36	4.24	<.001	3.02	8.52
Study Group X Timepoint	-2.14	2.01	-1.07	.292	-6.20	1.92
Gender (0)[c]	-4.82	2.00	-2.47	.021	-8.85	-0.78
Patient Relation (0)[d]	-3.11	1.99	1.56	.126	-0.91	7.13
Decision-Making Experience (0)[e]	-6.26	2.43	-2.57	.014	-11.17	-1.35
Perceived Stress	1.38	0.20	6.94	<.001	0.99	1.78
Random Effects						
Intercept	20.58	7.13		.004	10.43	40.59
Residual [Timepoint = 0]	22.12	7.33		.003	11.55	42.33
Residual [Timepoint = 1]	16.19	7.25		.026	6.73	38.93
Fixed Effects			Depressive Symptoms			
Intercept	21.47	5.47	3.93	<.001	10.46	32.49
Study Group (0)	4.27	2.28	1.87	.068	-0.34	8.87
Timepoint (0)	4.63	1.29	3.58	<.001	2.02	7.25
Study Group X Timepoint	-4.06	1.90	-2.13	.039	-7.91	-0.21
Gender (0)	-3.49	2.14	-1.63	.110	-7.80	0.82
Patient Relation (0)	1.76	2.13	0.83	.414	-2.54	6.05
Decision-Making Experience (0)	-2.82	2.60	-1.09	.283	-8.06	2.41
Perceived Stress	1.36	0.21	6.40	<.001	0.93	1.79
Random Effects						
Intercept	26.55	8.18		.001	14.51	48.56
Residual [Timepoint = 0]	19.84	6.94		.004	9.99	39.39
Residual [Timepoint = 1]	14.14	6.69		.035	5.59	35.74

[a] 0 = Usual Care (UC), 1 = Reappraisal-Enhanced Foundation for Regulating Affect and Managing Emotions (REFRAME).

[b] 0 = baseline, 1 = 5–6 days post-baseline.

[c] 0 = Man, 1 = Woman.

[d] 0 = spouse/partner, 1 = other.

[e] 0 = no, 1 = yes.

Table S3. Estimated marginal means (EMMs) for psychological distress severity across study groups and timepoints in a nonrandomized pilot trial of REFRAME, a tablet-based cognitive reappraisal intervention for surrogate decision-makers of critically ill patients (Cleveland, Ohio, 2023) (N = 48)

Effect	EMM	SE	95% CI	
			LL	UL
		Anxiety Symptoms		
Group				
UC[a]	61.84	1.76	58.29	65.38
REFRAME[b]	60.88	1.33	58.19	63.56
Timepoint				
T0[c]	63.71	1.33	61.04	66.38
T3[d]	59.01	1.34	56.31	61.70
Group X Timepoint				
UC X T0	63.65	1.91	59.80	67.50
UC X T3	60.02	1.89	56.21	63.84
REFRAME X T0	63.77	1.46	60.83	66.70
REFRAME X T3	57.99	1.53	54.91	61.08
		Depressive Symptoms		
Group				
UC	55.14	1.89	51.34	58.94
REFRAME	52.90	1.43	50.03	55.78
Timepoint*				
T0	55.32	1.40	52.50	58.14
T3	52.72	1.41	49.88	55.56
Group X Timepoint*				
UC X T0	55.43	2.02	51.37	59.49
UC X T3	54.85	2.00	50.83	58.88
REFRAME X T0	55.22	1.53	52.13	58.30
REFRAME X T3	50.59	1.60	47.38	53.80

[a] Usual Care.

[b] Reappraisal-Enhanced Foundation for Regulating Affect and Managing Emotions.

[c] Baseline.

[d] 5–6 days post-baseline.

This is a multimedia appendix to a full manuscript published in JMIR Formative Research. For full copyright and citation information, see http://dx.doi.org/10.2196/jmir.73769
